# What Is a Dingo? The Phenotypic Classification of Dingoes by Aboriginal and Torres Strait Islander Residents in Northern Australia

**DOI:** 10.3390/ani10071230

**Published:** 2020-07-20

**Authors:** Victoria J. Brookes, Chris Degeling, Lily M. van Eeden, Michael P. Ward

**Affiliations:** 1 Sydney School of Veterinary Science, Faculty of Science, The University of Sydney, Camden 2570, Australia; michael.ward@sydney.edu.au; 2 School of Animal and Veterinary Sciences, Faculty of Science, Charles Sturt University, Wagga Wagga 2650, Australia; 3 Australian Centre for Health Engagement, Evidence & Values, School of Health and Society, Faculty of the Arts, Social Sciences, and Humanities, University of Wollongong, Wollongong 2252, Australia; degeling@uow.edu.au; 4 School of Life and Environmental Sciences, Faculty of Science, The University of Sydney, Camperdown 2006, Australia; lily.vaneeden@sydney.edu.au

**Keywords:** dingo, Australia, phenotype, hybridisation, biosecurity, rabies

## Abstract

**Simple Summary:**

Dingoes are an Australian icon with cultural, as well as ecological, value, yet defining a dingo is complicated by hybridisation with domestic dogs. Northern Australia is a high-risk zone for the arrival of rabies, a disease which affects dogs (including dingoes) and people. In a rabies outbreak, Aboriginal and Torres Strait Islander peoples who live in this region would want dingoes protected. We visited the Northern Peninsula Area (NPA), Queensland, in 2018–2019 and surveyed Aboriginal and Torres Strait Islander residents about how they define dingoes, using pictures from camera traps previously placed and operated in the area. We found that dingo definition was based on characteristics traditionally associated with the iconic dingo (medium to large-framed dogs, with a long nose, pointed ears, narrow abdomen, bushy tail, smooth tan coats, and white feet and tail tip) but hybrid features, such as curled tail or a lack of white points, were also acceptable features. Local definitions are important when designing and implementing management plans so that actions are supported by local communities, and our findings are a useful guide for identifying dingoes in the NPA so that, in the event of a rabies outbreak, locally valued dingoes could be identified and protected.

**Abstract:**

Dingo classification and management is complicated by hybridisation with domestic dogs. Northern Australia is a relatively high-risk zone for a rabies incursion, and in the event of an incursion, Aboriginal and Torres Strait Islander peoples who reside in this region would prioritise the protection of dingoes. Therefore, the classification of dingoes in this context is important. Twelve pictures of canids with features associated with both dingoes and domestic dogs from camera traps in the Northern Peninsula Area (NPA), northern Queensland, were shown to Aboriginal and Torres Strait Islander rangers (n = 3), biosecurity officers (n = 2), environmental health workers (n = 2), and residents (n = 39) in the NPA. Nearly all pictures (10/12) were classified as dingo or domestic dog (none as hybrid) and two were inconclusive (no overall agreement). Dingoes were consistently identified as medium to large-framed dogs, with a long nose, pointed ears, narrow abdomen, a bushy or feathered tail, and smooth coats of a single base colour. Some hybrid features were acceptable, including sable coats, lack of white tail tip or feet, and curled tail. These findings are a preliminary guide for identifying canids in the NPA region for whom management might be controversial. Building on this approach via further consultation with residents is needed to inform rabies response policy. Our approach using locally acquired camera trap pictures could also be extended to other regions in which dingoes have value but their management is controversial.

## 1. Introduction

Understanding how people classify wild animals is an essential first step in determining the values which people assign to a wild animal, and is critical to planning and implementing conservation or management strategies [1]. Decision-making can become problematic in situations in which wild animals do not fit easily into Western classification systems—for example, in populations in which genetic material has introgressed from their domestic relatives. Management in such situations might require us to consider factors other than genetics when defining these populations in the wild—such as morphology, and their ecological and cultural roles—to determine how these animals are classified and, hence, assigned a value by human societies.

Management of the dingo in Australia is an example in which scientific classification causes controversy. Dingoes are variously referred to as *Canis dingo*, *C. lupus dingo*, and *C. familiaris* due to disagreements over their taxonomy and relationship with domestic dogs (*C. familiaris*), with whom they can interbreed [2,3,4]. Disagreement and ambiguity surrounding the dingo’s classification is reflected in policy and management in Australia, with both the protection and persecution of dingoes promoted or legislated [5,6]. In the context of livestock protection, dingoes are included with feral dogs and dingo–dog hybrids under what is typically termed, “wild dog management”, because distinguishing between these canid types based on morphological features is difficult [7]; consequently, management in this context generally does not seek to do so [8]. However, there are management contexts that require a more nuanced classification; a recent study in Northern Australia found that, in the event of an incursion of rabies, residents in remote communities would want dingoes protected [9]. As such, failure to understand which dogs are valued by these communities could erode trust in management authorities and prevent an effective response to a rabies outbreak. It is therefore important to understand how residents in remote communities define dingoes.

Northern Australia is considered a high-risk zone for a canine rabies incursion due to its abundant domestic dog populations in remote communities, dingo populations, its proximity to rabies-endemic Southeast Asia, and the recent spread of rabies in Indonesia [10,11,12]. A modelling study predicted that, should a rabies incursion occur, the probability of an epidemic in dingoes is 21%, most likely leading to endemicity without control interventions [13]. The nationally agreed policy for rabies control [14] states that if rabies is detected, susceptible populations need to be identified at the earliest possible time, including wild and free-roaming populations, so that appropriate, population-specific control strategies can be considered. In the case of wildlife, experts will be consulted to determine such strategies, which might include targeted vaccination. As stated in the current policy document [14], limited population reduction could also be considered on a case-by-case basis and could include both wild and feral animal populations.

In remote Indigenous communities in Northern Australia, most domestic dogs roam freely [15,16] and, therefore, can come into contact with dingoes. Hybridisation between dingoes and community-owned dogs in Northern Australia has been demonstrated [17]. The level of introgression in dingo populations is varied: it has been suggested to be widespread and increases in proximity to human habitation [4], but there are also regions in which it is still low [18]. Whilst there has been extensive research on the impact of dingoes and dingo–dog hybrids on ecosystems and livestock industries [19,20], and evidence that the Australian public values dingoes [21], little is known about how the Australian public perceives concepts such as hybridisation to identify and distinguish dingoes from other canids. Although morphometric measurement and phenotypic characteristics have been used to assess dingo purity [22], more recently, dingo definition has been based on genotype following analysis of DNA microsatellite loci [4]. These methods are not conclusive—for example, there is debate about relevant microsatellites and variation due to differing analyses, and phenotypic characteristics do not necessarily reflect the level of introgression. They also do not account for people’s contextual definitions of a dingo and their acceptability of hybridisation.

In this study, we explored how dingoes are defined by residents in remote Australian Indigenous communities in Northern Australia for the purposes of management to reduce disease risk, focusing on the Northern Peninsula Area (NPA) of Queensland. We specifically aimed to provide a preliminary characterisation of NPA residents’ definition of a dingo to inform Australian rabies incursion policy. We acknowledge parallels between the imposition of such classificatory systems and colonial practices of Indigenous assimilation [23,24]. However, our aim is that the findings of this study could provide foundations for in-depth community consultation to determine local preferences for dingo and dog management. More broadly, we demonstrate how using camera trap pictures could be used to increase the understanding and appreciation of the socio-cultural value that people assign to dingoes.

## 2. Materials and Methods

Three visits were made to the study region in 2018 and 2019. The study methods were approved by the Human Research Ethics Committee at the University of Sydney (Project No: 2018/451) and all methods were carried out in accordance with relevant guidelines and regulations.

### 2.1. Study Region

**Figure 1:**
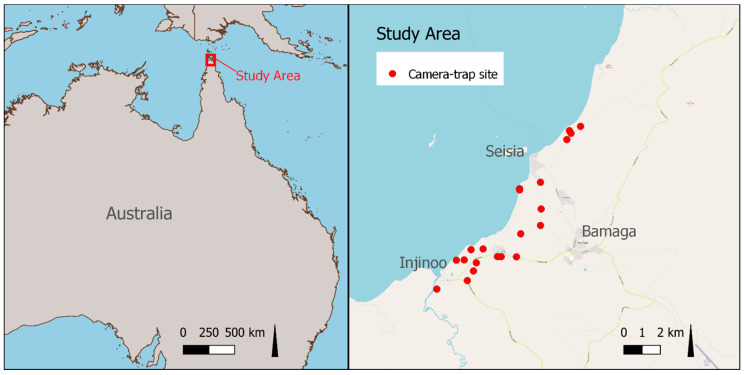
The study area (Northern Peninsula Area) location and camera trap sites, in northwestern Cape York, Queensland, Australia.

The Northern Peninsula Area is a local government area in northwestern Cape York, Queensland (Figure 1). It covers a total land area of 105,207 hectares and incorporates the communities of Bamaga, Injinoo, New Mapoon, Seisia, and Umagico (total population 2796, of whom 87% are Aboriginal and Torres Strait Islander people [25]). Vegetation coverage includes woodlands, grasslands, tropical rainforests, heathlands, wetlands, and river systems, of which over 99% is “remnant” vegetation (vegetation on land that has never been cleared by people) [26]. The NPA is adjacent to the Jardine National Park and the Great Barrier Reef which is a World Heritage listed site [27]. To the north lies the Torres Strait and Thursday Island, a principal administrative and transportation centre in the region.

### 2.2. Discussions with Stakeholder Groups

**Figure 2:**
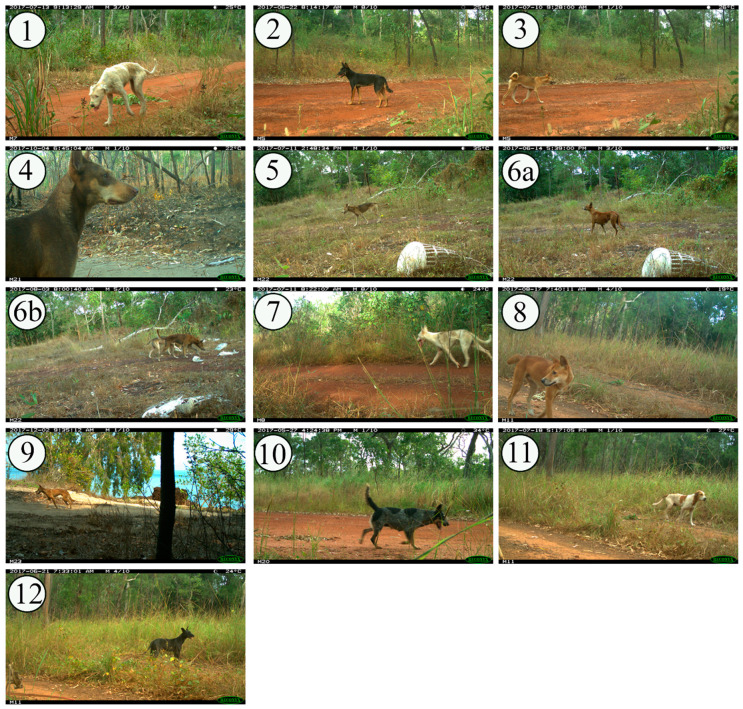
Pictures used in a study of the phenotypic classification of dingoes by Indigenous residents in northern Queensland, Australia.

We initially collected background information to develop a questionnaire for community residents. We (VB, CD, MW) had existing relationships with relevant stakeholder groups in the study region (Land and Sea Rangers, biosecurity officers, and environmental health workers (EHWs)), developed over the previous 6 years through research focused on preparedness for a rabies incursion [9,28,29,30,31,32]. Members of these stakeholder groups who were community residents were selected purposively because they were considered likely to have a high level of knowledge about dogs and dingoes in the NPA due to their work roles. Group members were shown 12 pictures of dogs, all of unknown genotype, that demonstrated a range of phenotypes (observable characteristics of an individual; Table 1 and Figure 2), including those typically associated with the hybridisation of dingoes [22]. We discussed aspects of dingo phenotype, ecology, and behaviour that could be used to inform the questionnaire for community residents. Pictures of dogs and dingoes used in the study were selected from images captured by motion- and heat-activated cameras (Reconyx HC600 Hyperfire, http://www.reconyx.com.au/, accessed 22 February 2019) at sites within 5 km of NPA communities between May 2017 and May 2018 (Figure 1). This provided local context by demonstrating a range of phenotypes recently observed in the study region. Specific landmarks were excluded to reduce bias in the opinion of participants due to their experience with dingoes and dogs that were potentially recognisable based on location.

### 2.3. Questionnaire Implementation with Community Residents

Researchers developed a face-to-face questionnaire informed by discussions with the stakeholder group members (Section 2.1), in which participants who were community residents were asked to state whether each picture (n = 12) was a dingo, domestic dog, or hybrid. Participants in the questionnaire were not members of the stakeholder groups. Following consultations with the stakeholder group members, Picture 6b replaced Picture 6a (Table 1 and Figure 2) from the original set so that there was a balance of phenotypes and expected categories. Enrollment was by convenience, and participation was voluntary and by written, informed consent. A copy of the questionnaire is included in the Supplementary Materials. The proportions of classifications (dingo, domestic dog, or hybrid) for each picture were tabulated (including 95% confidence intervals) and plotted. Agreement over classification by community residents was estimated using Fleiss’ kappa statistic.

## 3. Results

### 3.1. Stakeholder Groups

Participants from stakeholder groups included Land and Sea Rangers (n = 3), biosecurity officers (n = 2), and EHWs (n = 2). All were residents who were Aboriginal or Torres Strait Islander people, male, and between 18–60 years old.

Picture 8 was the most readily categorised as a dingo by all stakeholder group participants, due to its tan-coloured coat, face shape, pointed ears, white feet and nose, and bushy tail, and was described as the archetype dingo totem associated with some NPA clans. Pictures 3, 5, and 9 were also unequivocally categorised as dingoes. Group members recognised the animal in Picture 3, with several commenting, “this one’s famous round here”. They stated that they see two or three dingoes with similar curly tails in the local area, and that curly-tailed dingoes live throughout the northern Cape York Peninsula and down the west coast. They thought that this feature was the result of breeding between wild dingoes and one or more domestic dogs in the 1990s. In contrast, group members stated that dingoes around Ussher Point (Jardine River National Park, eastern Cape York Peninsula) have tails “almost as straight as a normal dog”. The grey colouration of the dingo in Picture 5 was reportedly due to age—participants stated that the coat would have been tan when the dingo was younger. The red colouration of the animal in Picture 9 was also considered typical of dingoes in the NPA. Head, body, and tail shape were considered the typical defining features of the dingoes in Pictures 3, 5, 8 and 9.

Stakeholder group members used behaviour and apparent gait (as far as can be assessed in a still image) to categorise dingoes when phenotype was equivocal. Some said Picture 2 was a domestic dog due to its colour (black and tan) but when told that this dog was seen in the company of dingoes, they decided that he was most likely a hybrid but would be classified as a dingo in the NPA. Black-coated dingoes were considered relatively new in the region. Most group members were adamant that Picture 7 was a domestic dog but some were uncertain due to the way in which it appeared to be moving (fast, as if with a purpose). Location did not appear to be useful when classifying dingoes because both dingoes and unaccompanied (free-roaming) domestic dogs are seen in the bush.

Even though contextual features were removed from the pictures, some of the stakeholders drew on their knowledge of the local dingo and dog populations in explaining how to categorize some of the canids in the sample. For example, rangers told us that dingoes are “getting pretty brave” because they are seen more frequently along the roads around the communities than they were 2–3 years ago. They attribute this to an increase in waste food thrown from cars by residents. They also see dingoes feeding on turtle carcasses at the local refuse site, as well as horse carcasses or even foals whilst a mare is foaling. Despite this perceived increase in the frequency of dingo activity around communities, rangers also stated that dingoes are scared of domestic dogs, and all groups agreed that a dingo and a domestic dog are unlikely to be seen together.

All other pictures (1, 4, 6a, 10, 11, 12) were unequivocally categorised as domestic dogs due to floppy ears, patchy coat colour, or domestic breed appearance (Pictures 4 and 10 were recognised as a Kelpie and a Blue Heeler, respectively).

### 3.2. Community Residents

**Figure 3:**
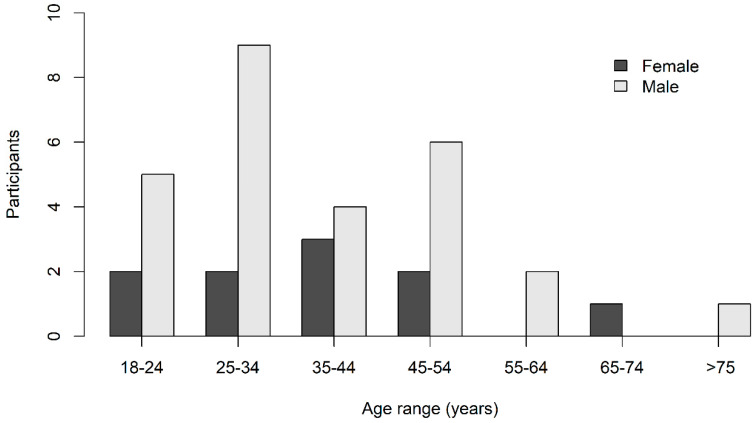
Age and sex of participants in a survey to explore how Aboriginal and Torres Strait Islander peoples define dingoes in the Northern Peninsula Area, Queensland.

A total of 39 residents participated in, and completed, the questionnaire survey. All age groups were represented and more male (73%) than female residents were surveyed (Figure 3). Participants represented all community groups present: Bamaga (n = 13), Injinoo (8), New Mapoon (8), Seisia (4), and Umagico (4).

**Figure 4:**
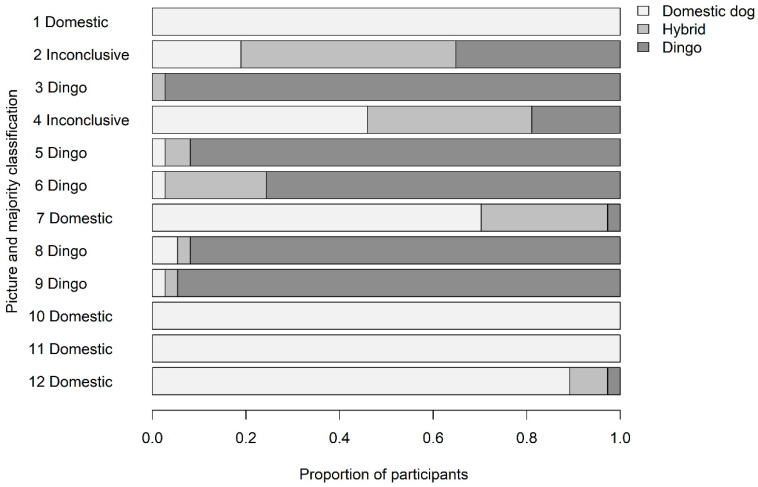
Proportions and majority classifications of 12 pictures of free-roaming canids by Indigenous residents, in a study of definitions of dingoes and domestic dogs in the Northern Peninsula Area, Queensland.

Overall agreement about picture classification between community residents was high (Fleiss’ κ = 0.62, *p* < 0.01, Figure 4). Participants used the term “house dog” rather than “domestic dog”, and the term “both” instead of “hybrid”.

Some pictures were unequivocally (Picture 3) or predominantly (Pictures 5, 6, 8, 9) classified as “dingo” by residents (Fleiss’ κ = 0.74 (*p* < 0.001) and the 95% CI of the proportion of residents who selected “dingo” did not overlap the 95% CIs of the proportions of those who selected “hybrid” or “domestic dog”, Table 2). Features that were consistent throughout these pictures included pointed ears, a long nose, medium size, light build with tucked-up abdomen, a brushy or feathered tail, and a single base coat colour (tan, red, or grey, with no colour patches). Features associated with hybridisation that were visible amongst these pictures included a curly tail, sable coat, and lack of white tail tip or white feet.

Some pictures were unequivocally (Pictures 1, 10, 11) or predominantly (Pictures 7, 12) classified as “domestic dog” by residents (Fleiss’ κ = 0.64 (*p* < 0.001) and the 95% CI of the proportion of residents who selected “domestic dog” did not overlap the 95% CIs of the proportions of those who selected “hybrid” or “dingo”; Figure 4). The dogs in these pictures all lacked some or all the features that were consistent in pictures identified as “dingo”. Instead, they included floppy ears, a short nose, broad cranium, larger frame and heavy build, thin tail, patchy coat colour, or obvious domestic breed type (Picture 10 was readily identified as the breed “Blue Heeler” by all residents).

Classifications of Pictures 2 and 4 were inconclusive. Although both dogs had features consistent with pictures classified overall as dingoes (consistent ear and head shape in both pictures, and body shape and tail in Picture 2), both dogs had bi-colour coats which are seen in the “Kelpie” breed of domestic dog.

Unlike the participants from the stakeholder groups, residents did not mention gait or behaviour when classifying dingoes and did not comment on the presence of the additional dog in Picture 6b. Instead, throughout all the pictures, they used ears, face, head, body, and tail shapes and coat colours to justify their choices.

Throughout all pictures, participants’ proportions of classifications as “dingo”, “hybrid”, and “domestic dog” were 0.42, 0.13, and 0.45, respectively.

## 4. Discussion

There are varied examples of how people in different Indigenous communities sometimes distinguish types of dogs, for example, as dingoes and domestic dogs, or “camp dogs” and “wild dogs” (reviewed in [6,33]), yet little has been reported about how these distinctions are made. In the current study, strong agreement about the types of dogs in pictures from camera traps demonstrated the characteristics that residents in the Northern Peninsula Area (NPA) of Australia consistently classified as belonging to dingoes, including their acceptance of some characteristics associated with hybridisation with domestic dogs. The residents’ opinions were consistent with those of the smaller group of stakeholders in animal and environmental health from the region (rangers, environmental health workers, and biosecurity officers).

It was apparent that respondents could base their definition of what a dingo is on phenotype and that this was generally sufficient to reach a majority opinion. Whilst we included one picture of a dog (Picture 8) in this survey that had the phenotypic characteristics that could be associated with a “pure” dingo [22,34], and members of one stakeholder group described this dog as the archetype dingo totem associated with some NPA clans, all respondents readily classified other dogs as dingoes, suggesting that NPA residents accept a broader range of canids into the “dingo” classification than scientists [22]. The constant morphological features of all pictures classified as dingoes were head and body shape and the size of the dog; all were pictures of medium- to large-framed dogs, with a long nose, pointed ears, tucked-up abdomen, and a bushy or feathered tail. Constants about coat were that all had smooth coats of a single base colour. Variable features included coat colour (tan, red, grey, and sable), white tips (not always present on all feet and tail), and tail shape (curly). These latter features are associated with hybridisation with domestic dogs [22].

Whilst this phenotypic approach to dingo classification in the NPA allowed for acceptance of some features of hybridisation, not all animals with the constant shape and size features associated with dingoes in this study were classified as dingoes. In these cases, other features associated with hybridization—including patchy coat colour, thin tail, and floppy ears—were present, and participants classified these as domestic dogs. Only two animals were inconclusively classified by residents, and disagreement appeared to be due to a coat colour that participants associated with a domestic breed (Kelpie) even though the body shape was compatible with a dingo. In this study, we did not investigate how the classification of dingoes changes with time and place, although discussion with stakeholder group members about the black and tan dog, as well as the dingo with the curly tail (with an accepted history of breeding with a domestic dog), suggested that that dingo status in the NPA is a fluid construct with acknowledged regional variation. Sightings of dingoes with black coats had only occurred recently in the NPA, and curly-tailed dingoes had appeared within living memory. Various coat colours, including black, have been documented historically in other parts of Australia [7], and also recently; for example, black and tan dingoes have been identified in the Tanami Desert [35]. In addition, Radford, et al. [36] found that dingoes in mountainous areas tended to be larger-framed than lowland dingoes. In the current study, behaviour and ecology played a role in classification by some stakeholder group members. It is possible that such factors might be influential in the acceptance of new dingo phenotypes in the NPA [37]; further investigation would be needed to understand how dingo classification evolves and varies between regions.

Residents rarely classified dogs in the pictures as hybrids and no dogs were majority classified as hybrids by residents. This might reflect the pictures selected for inclusion in the study, or it could reflect a reluctance of residents to use this term. The concept of hybridisation and its application in defining identity can be controversial beyond the sensitivities around managing an animal that has cultural value for these communities [33,38]. Arguments to maintain dingo “purity” mean that dingo–dog hybridisation is used as justification for destruction [39] (similar to how wolf–dog hybrids are culled in Europe [40]). However, this denies some wild canids the right to exist based on mixed ancestry that resulted from human actions; for example, the introduction of domestic dogs to Australia [6]. We acknowledge that, like most wildlife, dingoes have significance for Australian Indigenous cultures [2] that is largely overlooked in dingo management. As such, it is critical that decisions and implementation of the management of dingoes, especially where there is an attempt to distinguish between canids based on perceived ancestry, must be planned and conducted in consultation with Aboriginal and Torres Strait Islander peoples.

Managing interactions between scientific and Indigenous knowledge systems and understanding how dingoes are defined by local residents would be important during a rabies response in Northern Australia [41]. An effective biosecurity response requires a balance between the control of disease spread and the potential impacts of the disease, and needs consideration of the resilience of the populations impacted by the disease and the response so that strategies are accepted and supported. Resilience depends on a range of inter-linked systems, including society, culture, the economy, and the environment. Whilst we generally focus on response strategies for the control of disease spread given the potential impacts of the disease, for example, in the context of rabies in Australia [31,42], preparedness often ignores resilience and how this can be supported. Rather than waiting for an incursion, we suggest that Australia’s nationally agreed policy for rabies control [14] should incorporate the findings of this study as a preliminary assessment of dingo classification for the NPA and that preparedness activities should continue to build on them so that relevant populations are defined at response onset. We note that these findings are preliminary. The number of pictures used was limited, the number of residents was relatively small, and important questions remain for local residents—such as who ultimately decides that this approach to dingo classification is acceptable for this region and how. Further refinement and discussion about their acceptability and incorporation into policy needs to be undertaken with residents in the study region.

## 5. Conclusions

The results of this study focus on a pragmatic approach to classifying dingoes from the perspective of local residents so that dingoes can be identified and managed in a way that supports residents’ socio-cultural values during a biosecurity response to rabies in northern Queensland. In this way, local knowledge systems can be understood, which might otherwise be difficult to access. Indigenous residents in northern Queensland demonstrated agreement in their classification of dingoes and, although this is specific to this region, our approach could be used in other regions to expand understanding of how different communities classify dingoes. The results are preliminary and we note that community consultation is essential to build on these results. Decisions that could have social and cultural impacts require community consultation, otherwise trust in management authorities is eroded.

## Figures and Tables

**Table 1:** Characteristics of canids in pictures used in a survey to explore how Aboriginal and Torres Strait Islanders define dingoes in the Northern Peninsula Area, Queensland. The pictures are presented in Figure 2. Picture 6a was shown to participants from stakeholder groups, and Picture 6b was shown to community residents.

Picture	Head	Ears	Body Conformation	Coat	Tail Shape	White Points	Domestic Breed
1	Short nose, broad cranium	Floppy	Large frame, light build	Rough, patchy white-brown	Thin	NA	–
2	Long nose	Pointed	Medium frame, heavy build	Black and tan	Bushy	Back foot	–
3	Long nose	Pointed	Medium frame, light build	Tan	Curly and bushy	Front foot	–
4	Long nose	Pointed	NA	Brown, kelpie markings	NA	None apparent	Kelpie
5	Long nose	Pointed	Medium frame, light build	Sable, grey to light tan	Bushy	Yes	
6a	Long nose	Upright but not pointed	Large frame, heavy build	Red	Thin	Nose	–
6b	Long nose	Pointed	Large frame, light build	Red	Thin with feathering	All feet	–
7	Long nose	Pointed	Medium frame, light build	Patchy white and brown	Thin	NA	–
8	Long nose	Pointed	Medium frame, light build	Tan	Bushy	Yes	–
9	Long nose	Pointed	Medium frame, light build	Tan-red	Bushy	None apparent	–
10	Long nose	Pointed	Small frame, heavy build	Patchy black and grey with ticking	Bushy	None	Blue cattle dog
11	Short nose, broad cranium	Floppy	Medium frame, heavy build	Patchy brown and white	Thin	NA	–
12	Long nose	Upright but not pointed	Large frame, heavy build	Black	Thin	None apparent	–

**Table 2:** Proportion of respondents and 95% confidence intervals (“low”–“high”) that defined pictures as dingo, hybrid, or domestic dog in a survey of Indigenous residents in the Northern Peninsula Area, Queensland.

	Dingo			Hybrid			Domestic			
Picture	Proportion	Low	High	Proportion	Low	High	Proportion	Low	High	Overall Classification
1	0	0	0.05	0	0	0.05	1	1	1	Domestic
2	0.4	0.23	0.6	0.37	0.2	0.57	0.23	0.07	0.43	Inconclusive
3	1	1	1	0	0	0.05	0	0	0.05	Dingo
4	0.2	0.03	0.39	0.3	0.13	0.49	0.5	0.33	0.69	Inconclusive
5	0.93	0.9	1	0.07	0.03	0.17	0	0	0.1	Dingo
6	0.8	0.7	0.95	0.17	0.07	0.32	0.03	0	0.19	Dingo
7	0.03	0	0.21	0.27	0.13	0.44	0.7	0.57	0.87	Domestic
8	0.97	0.93	1	0	0	0.06	0.03	0	0.09	Dingo
9	0.93	0.9	1	0.03	0	0.13	0.03	0	0.13	Dingo
10	0	0	0.05	0	0	0.05	1	1	1	Domestic
11	0	0	0.05	0	0	0.05	1	1	1	Domestic
12	0	0	0.1	0.07	0.03	0.17	0.93	0.9	1	Domestic

## Supplementary Materials

The following are available online at https://www.mdpi.com/2076-2615/10/7/1230/s1: Questionnaire; questions in red were not used in the survey. Participants were asked questions and shown pictures, and surveyors completed the responses on the form.
