# Supplementary material for: What Is a Dingo? The Phenotypic Classification of Dingoes by Aboriginal and Torres Strait Islander Residents in Northern Australia
Source: Animals (Basel). 2020 Jul 20;10(7):1230. doi: 10.3390/ani10071230 (PMC7401616; doi:10.3390/ani10071230)
Supplement: Supplementary file 1 [file animals-10-01230-s001.pdf]

What is a Dingo? The Phenotypic Classification of Dingoes by Aboriginal and Torres Strait Islander Residents in Northern Australia

Victoria J. Brookes, Chris Degeling, Lily M. van Eeden, Michael P. Ward

Supplementary Material: Questionnaire; questions in red were not used in the survey. Participants were asked questions and shown pictures, and surveyors completed the responses on the form.

What is a dingo? Perspectives on dingo phenotype in northern Australia

Questionnaire Part 1

Name:

Sex:

Do you identify as an Aboriginal and/or Torres Strait Islander?

Do you have dingo or dog as a totem? Which one?

Age: 18-24 years
25-34 years
35-44 years
45-54 years
55-64 years
65-74 years
≥ 75 years

Do you work with animals or the environment (and duration, years)?

In which community do you currently live?

How long (years) have you lived in the NPA?

ID (for anonymization):

Question prompts for researchers:
What type of dog is in the picture? (Circle the answer).
If the answer is domestic or dingo: Is it completely X? (Mark the line).
If the answer is hybrid: Is it closer to a domestic or a dingo? (Mark the line).
If hybrid: If the council were making a policy to protect dingoes, would you count this dog as a dingo for protection?
What is it about this dog that made you decide how it should be classified? (Tick and add information)

Questionnaire Part 2 **ID……….**

1. Dingo ____________________H____________________ Domestic Dog
colour □ shape □ face □ ears □ feet □ tail □ gait □

2. Dingo ____________________H____________________ Domestic Dog
colour □ shape □ face □ ears □ feet □ tail □ gait □

3. Dingo ____________________H____________________ Domestic Dog
colour □ shape □ face □ ears □ feet □ tail □ gait □

4. Dingo ____________________H____________________ Domestic Dog
colour □ shape □ face □ ears □ feet □ tail □ gait □

5. Dingo ____________________H____________________ Domestic Dog
colour □ shape □ face □ ears □ feet □ tail □ gait □

6. Dingo ____________________H____________________ Domestic Dog
colour □ shape □ face □ ears □ feet □ tail □ gait □

7. Dingo ____________________H____________________ Domestic Dog
colour □ shape □ face □ ears □ feet □ tail □ gait □

8. Dingo ____________________H____________________ Domestic Dog
colour □ shape □ face □ ears □ feet □ tail □ gait □

9. Dingo ____________________H____________________ Domestic Dog
colour □ shape □ face □ ears □ feet □ tail □ gait □

10. Dingo ____________________H____________________ Domestic Dog
colour □ shape □ face □ ears □ feet □ tail □ gait □

11. Dingo ____________________H____________________ Domestic Dog
colour □ shape □ face □ ears □ feet □ tail □ gait □

12. Dingo ____________________H____________________ Domestic Dog
colour □ shape □ face □ ears □ feet □ tail □ gait □
